# A featureless approach for object detection and tracking in dynamic environments

**DOI:** 10.1371/journal.pone.0280476

**Published:** 2023-01-17

**Authors:** Mohammad Zohaib, Muhammad Ahsan, Mudassir Khan, Jamshed Iqbal

**Affiliations:** 1 National Center of Robotics and Automation (NCRA), Department of Mechatronics and Control Engineering, University of Engineering and Technology Lahore, Lahore, Pakistan; 2 Syed Babar Ali School of Science and Engineering, Lahore University of Management Sciences, Lahore, Pakistan; 3 School of Computer Science, Faculty of Science and Engineering, University of Hull, Hull, United Kingdom; Universiti Sains Malaysia, MALAYSIA

## Abstract

One of the challenging problems in mobile robotics is mapping a dynamic environment for navigating robots. In order to disambiguate multiple moving obstacles, state-of-art techniques often solve some form of dynamic SLAM (Simultaneous Localization and Mapping) problem. Unfortunately, their higher computational complexity press the need for simpler and more efficient approaches suitable for real-time embedded systems. In this paper, we present a ROS-based efficient algorithm for constructing dynamic maps, which exploits the spatial-temporal locality for detecting and tracking moving objects without relying on prior knowledge of their geometrical features. A two-prong contribution of this work is as follows: first, an efficient scheme for decoding sensory data into an estimated time-varying object boundary that ultimately decides its orientation and trajectory based on the iteratively updated robot Field of View (FoV); second, lower time-complexity of updating the dynamic environment through manipulating spatial-temporal locality available in the object motion profile. Unlike existing approaches, the snapshots of the environment remain constant in the number of moving objects. We validate the efficacy of our algorithm on both V-Rep simulations and real-life experiments with a wide array of dynamic environments. We show that the algorithm accurately detects and tracks objects with a high probability as long as sensor noise is low and the speed of moving objects remains within acceptable limits.

## 1 Introduction

Recent technological advancements in several multi-disciplinary domains engage mobile robots [1–3] to accomplish diversified tasks in various complex and challenging operational scenarios, thus highlighting the importance of accurate contextual characterization of both static and dynamic parts of the environment [4]. Typical applications of robots include executing pick and place tasks in warehouses [5–7], serving in restaurants [8], agriculture [9, 10], medical [11, 12], autonomous driving [13–16], Space [17, 18] and various industrial applications [19–22]. Most of these applications require environmental information to plan and follow the desired trajectories. Efficient path planning and collision avoidance essentially depend upon the surrounding information [23]. The critical role of environmental information can be exemplified by the unfortunate collision of the Google/Tesla self-driving car. Other self-driving car companies including Volvo, Bosch, Uber, Ford, and FiveAI have also reported similar navigation problems. By critically prioritizing human safety, future developments in mobile robotics are anticipated to minimize fatal accidents and encounters [24–26]. This is to be achieved by making the vehicle intelligent so that it can sense and handle uncertainties inherently present in the dynamic environment.

### 1.1 The challenge

The inevitable involvement of obstacles related to humans significantly elevates the complexity of profiling the dynamic environment for the navigating robot. However, if a robot has environmental information, including the position of static and dynamic obstacles, it can generate a safe trajectory. Global path planning allows the robot to generate a time-invariant path based on the position of static objects. However, the presence of dynamic objects requires run-time obstacle avoidance strategies through local path planning [27]. Moreover, with experience, the robot learns the nature of environmental changes and generates several possible local paths with respect to uncertainties, thus highlighting the significance of static and dynamic information [28]. Based on accurate surrounding information for autonomous navigation in unknown environments, the ultimate goal is to efficiently use sensory percepts to separate static and dynamic objects.

The critical problem is to extract the dynamic part of the environment from sensory data, similar to tracking objects in an unknown environment. In computer vision, object identification methods are classified into four categories; motion-based, model-based, appearance-based, and feature-based [29–32]. However, in the case of laser data, the appearance-based method may not be applied directly due to limited information. Model and feature-based approaches require prior knowledge, i.e., features and dimensions of the objects present in an environment. The dimensions can be predicted by estimating keypoints on the objects representing the geometrical features [33–35]. On the other hand, no prior information is needed for motion-based methods [36, 37]; these find application in urban environments wherein model-based techniques may fail against unforeseen scenarios. This may be considered as an advantage of the motion-based methods in comparison with the model-based counterparts.

### 1.2 Direction of the research

Considering the pivotal role of environmental information in autonomous navigation and the shortcomings mentioned above in related schemes, the present research aims to propose an algorithm for obstacle detection and tracking [23]. It should be able to identify static and dynamic obstacles separately while ensuring reliability. We adopt a motion-based approach that deploys a LiDAR sensor to track moving objects. Several dynamic environments are created and simulated in V-Rep. The algorithm is implemented in Robot Operating System (ROS) Melodic Morenia in Ubuntu version 18.04 environment on i7 laptop with 16 GB RAM. A 2D Sweep LiDAR sensor is used for real-world experiments.

The remaining paper is structured as follows. Section 2 presents the literature on existing approaches for object detection and tracking. The proposed algorithm for the separation and tracking of static and dynamic objects is described in Section 3. For validation, various scenarios are tested to verify the accuracy of the proposed algorithm. Simulation and experimental results demonstrating successful tracking of objects are presented in Section 4. Section 5 concludes the manuscript with potential directions for future work.

## 2 Literature review

The scientific community reports several techniques to solve the problem highlighted in Section 1.1. Schulz et al. presented an approach for human movement detection in an office environment using feature-based techniques [38]. The approach is suitable for the indoor environment. In contrast, an outdoor environment poses difficulty in feature extraction due to several similar objects in the same surroundings, i.e., a tree can be recognized as a pedestrian. Also, the proposed technique can only be used for objects with specific shapes. Some of the other detection methods generate an occupancy grid map and then classify the nature of cells as static and dynamic [39]. Generally, such techniques suffer from problems like wrong velocity estimation, occlusions, and corrupted object silhouettes. Wang et al. also utilized occupancy grid maps for moving objects detection [40]. However, they adopted the background subtraction method to suitably monitor the motion of an object. However, the proposed method is incapable of detecting the static or dynamic nature of the first detected obstacle. This technique is also known as scan-matching that compares two consecutive scans after best alignment [41, 42]. Another work characterizing the nature of objects as a separate static map and a dynamic map is reported in [43]. However, it is not obvious how the objects have been extracted from the dynamic maps. Petrovskaya and Thrun proposed a model-based approach to detect moving objects. This research uses pre-modelled objects and detects these using the polar coordinate and scan differencing method. The presented method detects vehicles; however, it does not recognize unknown objects like pedestrians, bicyclists, etc. [44]. A similar model-based approach is proposed by Vu & Aycard in [45]. They introduced several fixed models that can be used to classify pedestrians, bikes, cars, or buses. Simultaneously Localization And Mapping (SLAM) is considered a solved problem for static environment [46]. However, it has not been fully explored for dynamic environments.

Several motion-based tracking algorithms have been under consideration that utilize Artificial Intelligence (AI) inspired techniques. For example, the Kalman or particle filter can be used in order to estimate an obstacle’s position [47]. A probabilistic method, the Bayesian network can be used to generate two occupancy grid maps for presenting static (*p*(*S*^*t*^|*o*^1^…*o*^*t*^)) and dynamic (*p*(*D*^*t*^|*o*^1^…*o*^*t*^)) objects separately [48]. Where *S*^*t*^ and *D*^*t*^ represent static and dynamic maps at time *t*, respectively. The *o*^*i*^ is the *i*^*th*^ observation. The method is suitable for object separation; however, it does not maintain any history of the previous occupancy of the cells in the dynamic map (*D*). The information in the map only needs to be set in order to represent the current occupancy of each cell.

## 3 Research methodology

Keeping in view the aforementioned literature, we are interested in a motion-based approach for object detection using only 2D laser data containing the depth of the obstacles. Inspired from the Bayesian method [48], the presented research introduces an approach to separate and track moving objects. In order to address the limitations of the method [48], our methodology keeps a history of the objects from the beginning. An object detected by the LiDAR sensor consists of several data points. The monitoring (comparison, detection, or identification) of the object using data points is an intensive task [49]. Therefore, a suitable option is to create a cluster of nearest data points, such that each cluster represents a separate object. Consequently, we need to monitor only a cluster in order to track an object. It significantly reduces the complexity and increases the efficiency of the algorithm. For this purpose, sensory information is transformed from laser data (in the local frame) to Point Cloud XYZ (PC-XYZ) in the global frame. The conversion is achieved by calling different ROS nodes. The converted data points are then clustered using a Euclidean distance based approach. The approach, with the help of parameters (minimum/maximum points for clustering and tolerance), defines the environmental objects. Where, the tolerance is the radius for the k-NN searching, used to find the candidate points for a cluster. All the points within the radius are clustered and considered as a single object. We selected the radius based on several trials. We observed that in the case of a large radius, multiple objects are considered as a single object, whereas, for a small radius, a single object is considered as multiple objects. We found that 5cm is an optimal radius for our experiments. It can be updated with respect to the environment—the size of the objects, how close they are to each other, etc. Moreover, We consider minimum 15 and maximum 5000 data points. Data points fewer than 15 are considered as noise and are discarded. However, the upper limit can be updated corresponding to the dimension of an object.

**Fig 1:**
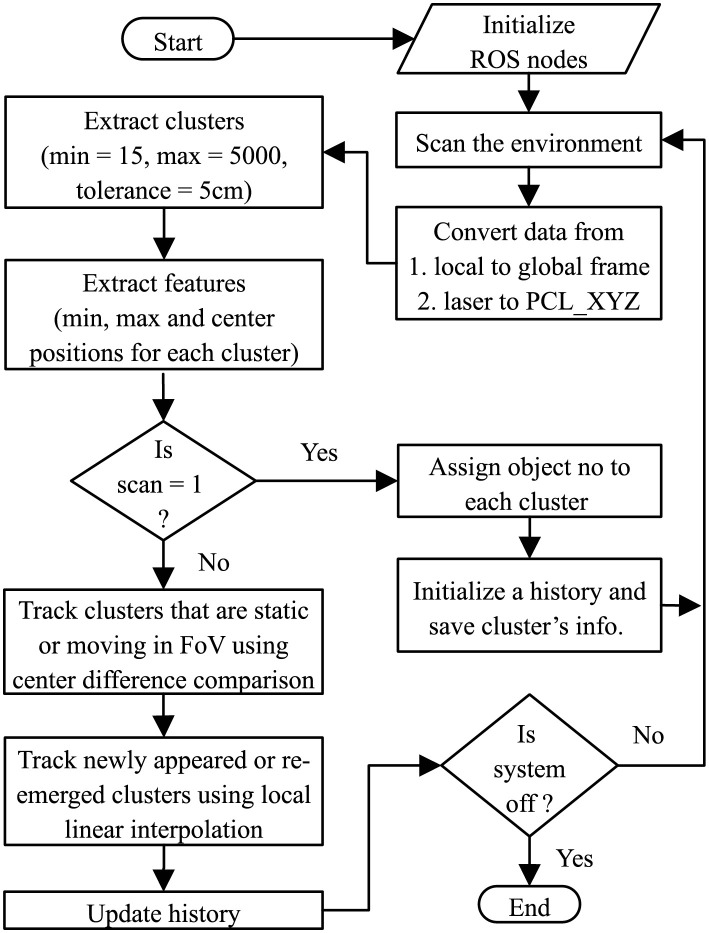
Flowchart of the proposed approach presenting a step-by-step process of tracking the objects by maintaining the history.

After object detection, the next vital step is to identify these and track their movement. Object identification is achieved by finding the dimensions (center, minimum, and maximum points) of each cluster. We utilize the cluster’s parameters for calculating the average in order to get the required dimensions with the help of the Point Cloud Library (PCL) feature extractor. We create a boundary box around every cluster for ease in visualization. The parameters of the cluster are stored in the history along with the cluster number and its status (i.e., moving, static, or outside the Field of View (FoV)). Initially, all the detected objects are considered as static and are stored in the history with random object numbers. However, in corresponding scans, the objects are tracked as static or dynamic using center difference comparison. Whereas the objects re-emerging from outside FoV are estimated by incorporating local linear regression. The process of objects detection, identification, and tracking in a dynamic environment is illustrated in Fig 1.

**Fig 2:**
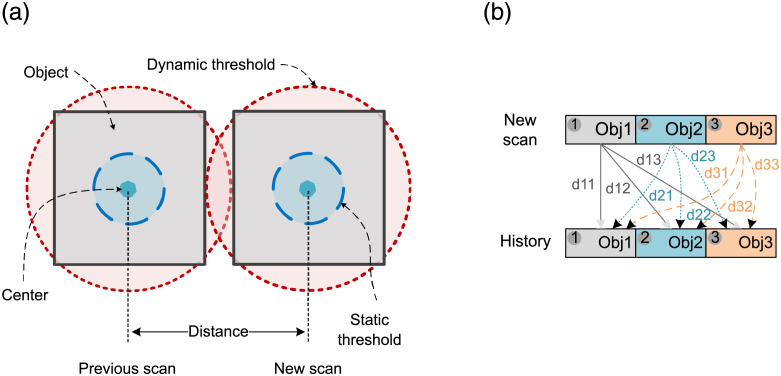
Identification of static and moving objects inside FoV.

The first step is related to the identification of the objects that are static or moving in FoV. This is achieved by defining two thresholds (static and dynamic) around each detected object. Fig 2(a) illustrates the thresholds for a square-shaped object. It can be observed that the static threshold is too small with respect to the size of the object. Therefore, it minimizes the incorrect estimation, i.e., two objects cannot exist inside the static threshold. Similarly, it also endures the sensory noise, i.e., the center point of a cluster can vary within the region. The dynamic threshold is selected to be greater than the rotational radius of objects with an assumption that the objects are of the same size. However, it can be adjusted by considering the object’s size and speed. So, it is large in size for speedy objects and short for slow-moving objects. If *R*_*obj*_ is the object’s rotational radius, *V*_*obj*_ is its velocity and *W*_*obj*_ is the object’s width, the radius of the dynamic threshold ($R_{dy_{threshlod}}$) can be determined by (Eq 1).

$$\begin{array}{c} R_{dy_{threshlod}}=\frac{V_{obj}\times 3W_{obj}+R_{obj}}{3W_{obj}}. \end{array}$$ (1)

Considering the limitations of a physical environment, it is assumed that $R_{dy_{threshlod}}$ can vary from static threshold to 3 × *W*_*obj*_. The objects detected in the new scan are considered one by one for comparison with all the objects available in the history, as shown in Fig 2(b). The top and bottom rows, presenting three objects, correspond to the new scan and history, respectively. The *d*_*ij*_ in the figure is the measured distance of the detected object *i* from object *j* of history.

If the distance *d*_*ij*_ lies within the static threshold, the object *i* is considered as static object *j*. This presents a slight change in the object’s position. Likewise, if the calculated minimum distance is observed between static and dynamic thresholds, the object is considered as dynamic. These detected static and dynamic objects are saved in the history with appropriate original objects. At this stage, history is updated for the tracked static and moving objects only.

**Fig 3:**
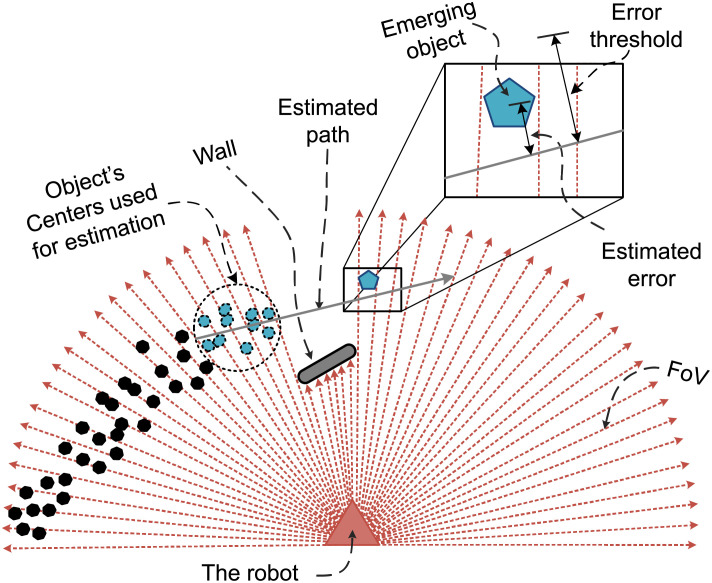
Estimation of objects using local linear regression.

In the second step, we consider the objects that disappeared in the last scan(s) and are re-emerged in the FoV (occlusion scenario). These objects and any other newly available untracked objects are estimated using local linear regression as illustrated in Fig 3.

The arrows emerging from the robot represent the sensor’s FoV. A wall in front of the robot limits the FoV. The tiny dots on the robot’s left side symbolize the positions of an object saved in history. The dots enclosed by the circle shown on the left side of the wall are the recently observed positions of the object. The scenario illustrates that the object is moving from left to right towards the wall. After some scans, the object is observed very close to the wall, and finally, it moves behind the wall. In these scans, the robot cannot detect the object as it is out of its vicinity. The status of the object in the history is set to an unobservable object (hidden state). After some scans, the same object appears on the right side of the wall. At this stage, the approach uses the previous ten histories of the object and calculates the local linear regression line using (Eq 2).

$$\begin{array}{c} \left[m,c\right]={\left(x^{T}\cdot x\right)}^{-1}\times x^{T}\cdot y\\ x=[\begin{array}{cc} x_{1} & 1\\ x_{2} & 1\\ x_{3} & 1\\ \vdots & \vdots \\ x_{10} & 1 \end{array}],y=[\begin{array}{c} y_{1}\\ y_{2}\\ y_{3}\\ \vdots \\ y_{10} \end{array}] \end{array}$$ (2)

Where *x* and *y* represent the positions of the object as per the last ten histories. The *m* and *c* are the slope and y-intercept of the regression line, respectively. After approximation, a tangent distance of the object’s new position from the line is measured. If the distance is within the error threshold, the object is considered as the corrected estimated object and is mapped to the previous original object in the history. The error threshold is set equal to the static threshold. If multiple objects move outside the FoV in a single scan, then a separate regression line is calculated corresponding to each object. On reappearance, the distance of each object is measured with every regression line one by one. If an object is found comparatively closest to the regression line with a distance below the error threshold, it is considered as the corresponding line object. However, if an object corresponds to more than one line, it is skipped and the next scans are awaited since if the object is dynamic, it will move and consequently will provide a better clue for its estimation. However, if an object appears to be static in several consecutive scans, it is considered as a newly appeared static object.

In the third step, we focus on the objects that do not lie in the region of any regression line. Such objects are also considered as newly detected objects and are added directly to the history as new entries. Table 1 presents the process of tracking those objects that are observed outside the FoV in some scans using estimated regression lines. A and B represent the number of nearer and far lines, respectively.

**Table 1:** Object tracking using local linear regression.

A	B	Action for objects	Remarks
0	0	Skip	The object must be either near or far from any line
0	1 or > 1	Add in history as new entry	Newly appeared object is found
1	x	Add in history with estimated object	Object is correctly found and updated in history as original
>1	x	Skip	Wait for next scans

The history contains objects (*Obj*_*i*_) with respect to scan numbers (scan *j*). Where each object has its unique number, status (static or dynamic), center, minimum and maximum point. Although the objects are not tracked in sequence, they are appropriately mapped in the history. The simulated results of the algorithm are discussed in Section 4.

## 4 Results

This section validates the proposed approach by presenting accurate results in different scenarios. One of the test cases is inspired from a real-world example of an agent tracking a flock of birds. The goal is to achieve approximately zero relative velocity of the agent w.r.t. the flock. Analogously, the agent robot detects, tracks and follows the remaining robots in the environment. To validate the approach in a real-world environment, experiments are conducted in which a human is moving in a room in an irregular path. Finally, by considering the selected thresholds, boundaries of the proposed approach are discussed w.r.t. the speed and turns taken by the objects inside or outside FoV.

### 4.1 Simulation results

The simulated environments with different configurations are created by considering static and dynamic objects where the dynamic objects follow linear, angular or irregular trajectories. The results show that the hidden objects are correctly tracked after being visible to the robot.

#### 4.1.1 Environment having a dynamic object hidden from the robot’s FoV

**Fig 4:**
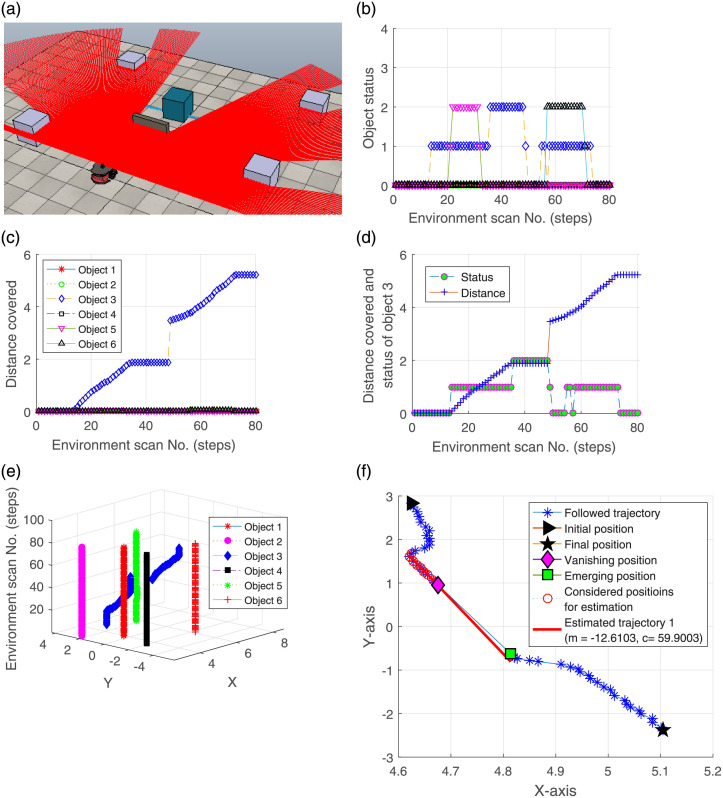
An experiment presenting tracking of a dynamic object in occlusion case.

An environment is created by introducing a wall between the robot and the dynamic object. The object starts moving inside FoV before being occluded by the wall. The robot successfully tracks it using the local linear regression on its reappearance. The environment highlighting the occlusion scenario of a dynamic object in the presence of multiple static objects is illustrated in Fig 4(a). The status of the objects and the distance covered by them from their emerging initial positions are illustrated in Fig 4(b) and 4(c), respectively. In Fig 4(b), 0 on the y-axis indicates static, 1 represents moving, and 2 shows the invisible state of the objects. It can be visualized from the status plot that three objects (3, 5 and 6) are invisible to the robot in different scans. However, Fig 4(c) validates that only object 3 covers some distance and objects 5 and 6 remain stationary throughout the scans. They are hidden in some scans due to the movement of the dynamic object (Obj 3). The rest of the objects (1, 2 and 4) are detected as static. The tracking performance of object 3 is illustrated in Fig 4(d). It is clear that after being invisible in scan 35, the object re-emerges in scan 48. A 3D view of the trajectories followed by the objects is depicted in Fig 4(e). The gap in the trajectory of object 3 highlights that the object information is not present in the history profile and it is in the invisible states (i.e. 35 to 48). Similarly, the connectivity line also validates the tracking of object 3. The approximation of the line and tracking of the dynamic object (as detailed in Section 3) is depicted in Fig 4(f). The vanishing and emerging positions of the object are illustrated. The approach estimates object 3 by considering its previous history and tracks it correctly. After reaching the final position, the object becomes static, which can be noticed in the figure.

The results verify that the dynamic object is successfully separated and tracked on its re-emergence in FoV after being hidden in the presence of multiple stationary objects. Although, due to occlusion, some objects are detected at different positions after some time, however, the proposed approach follows the objects previous history instead of considering these to be new objects, thus highlighting its novelty.

#### 4.1.2 Environments containing objects with nonlinear, irregular and parallel trajectories

**Fig 5:**
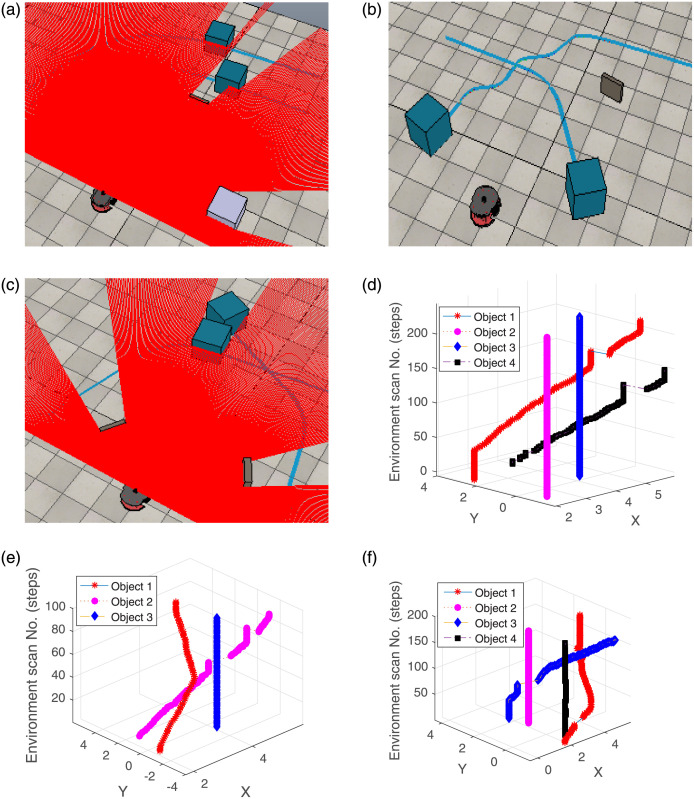
Accurate tracking of objects following nonlinear, irregular, parallel and crossing trajectories.

Three different scenarios are discussed here. Fig 5(a) illustrates a nonlinear parallel motion of the two objects in the presence of a static object and a wall. Initially, three objects are detected, i.e., a dynamic object (Obj 1), a wall and a static object. However, a second dynamic object goes hiding behind the first dynamic object. After some time, due to the long trajectory, the second dynamic object becomes visible to the robot and is considered as object 4. During the locomotion, both the dynamic objects disappeared due to the wall and are then re-emerged in a very close span. By considering the previous history of the objects, object 1 and 4 are correctly estimated after their re-emergence in the robot FoV as highlighted by the trajectories in the 3D plot (Fig 5(d)). Fig 5(b) shows objects exhibiting irregular and intersecting trajectories. The corresponding result of the objects tracking is illustrated in Fig 5(e). Both objects are visible to the robot. One object moves in a nonlinear fashion while the other follows an irregular trajectory in the beginning. Both objects are detected and tracked accurately. Another similar scenario is illustrated in Fig 5(c), where two objects pass each other within close proximity. Both the objects are detected only once. Object 1 disappears twice; however, it is found to be stationary after the second emergence. Therefore, the second estimation is not required. A 3D view of the followed trajectories presenting accurate tracking is depicted in Fig 5(f). The objects in all the environments are successfully tracked accurately, demonstrating the algorithm’s robustness.

#### 4.1.3 Special scenario—Following birds flock

**Fig 6:**
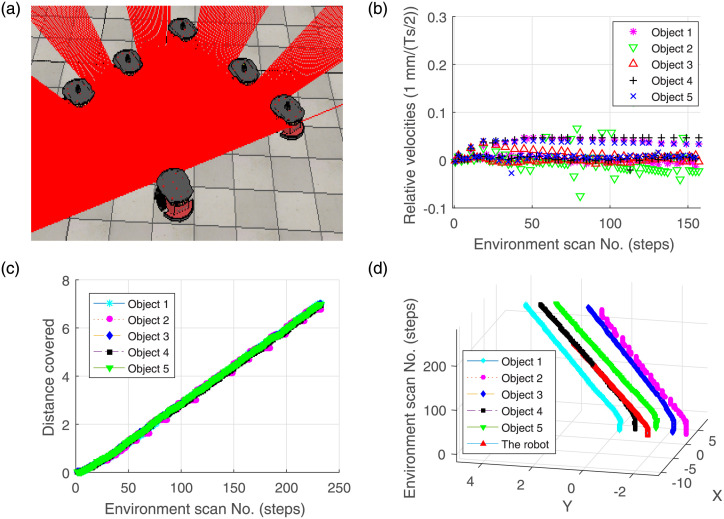
Validation results in the case of birds flock scenario.

A scenario “following the birds flock” further highlights the accuracy of the approach by considering zero relative velocity between bird robots and an agent robot. In Fig 6(a), the agent robot moves but remains stationary with respect to the birds flock. The velocities are compared in Fig 6(b). The estimated distance covered by the bird robots and trajectories followed by the birds and the agent robot are presented in Fig 6(c) and 6(d), respectively.

The above results demonstrate that the proposed algorithm is potentially helpful in the tracking of parallel moving robots, swarm robots, collaborative robots [50] etc.

### 4.2 Experimental results

**Fig 7:**
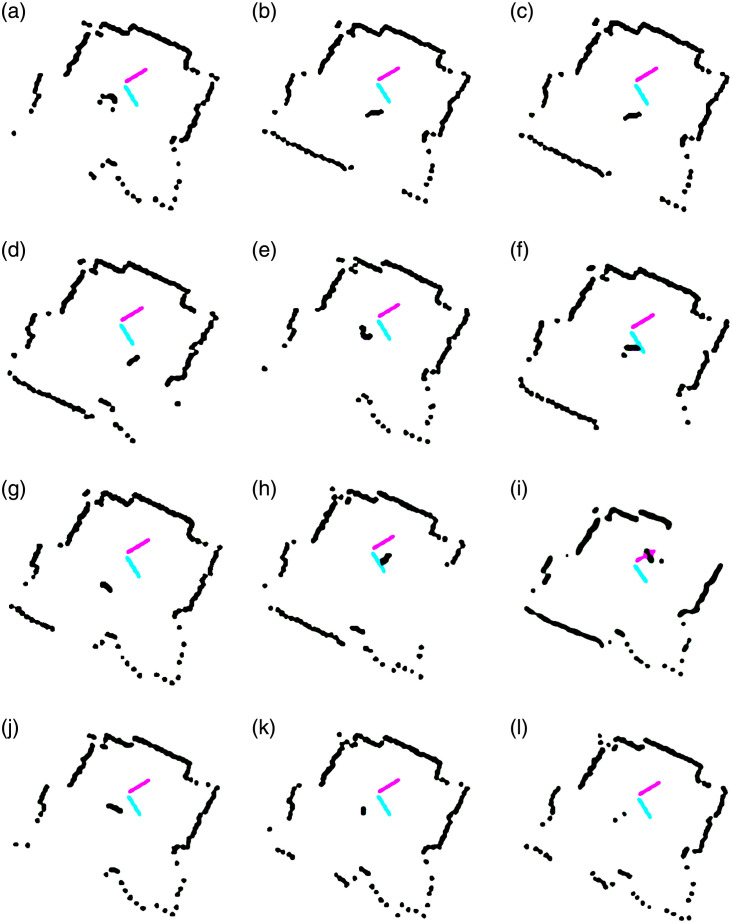
Human moving in a room (a to l).

After successfully demonstrating the efficacy of our algorithm in the simulation environment, we now validate its real-life applications through experimental results. The experiment setup contains a Scanse Sweep LiDAR sensor integrated with a four-wheel robot. The sensor is capable of scanning in 360° with a 1cm resolution and has a default sampling rate of 500Hz (which can be increased to 1075Hz). It can detect objects within the 40m range. The detected walls and a human walking randomly in a room are shown in Fig 7(a)–7(l). It can be observed that in most cases, the human is detected by the LiDAR sensor. However, due to hardware imperfections and sensor performance limitations, it occasionally fails to capture human being full motion (Fig 7(k) and 7(l)). Overall, experimental results establish that the algorithm not only correctly distinguishes between the stationary objects i.e., walls and the moving object i.e., the walking person, but also accurately tracks the latter.

**Fig 8:**
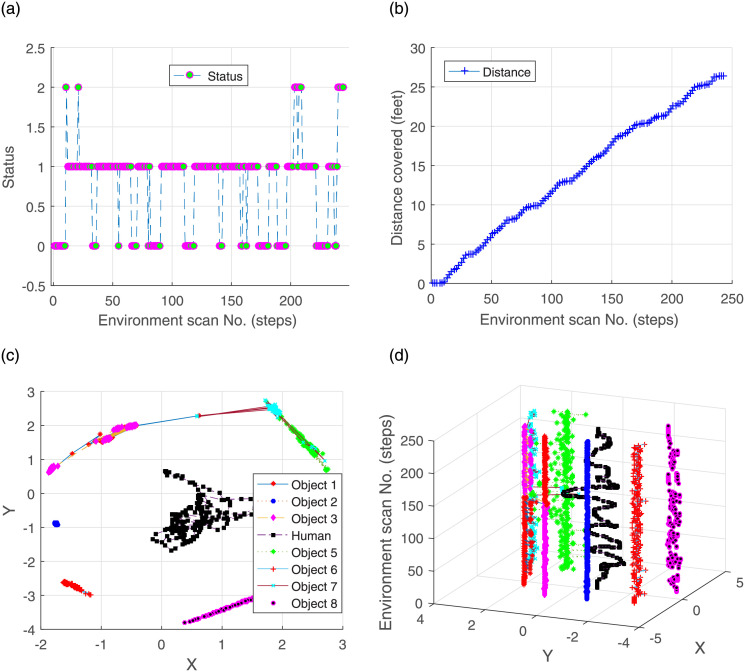
Tracking of a human moving inside a room.

The status and distance covered by the human are illustrated in Fig 8(a) and 8(b), respectively. The static position is indicated with ‘0’ status on the y-axis. The status ‘2’ shows that the object is invisible to the robot. Most of the time, the status remains ‘1’, indicating human locomotion. Another performance metric is an estimated location. The Fig 8(c) attributes motion to walking human only; the walls are treated as stationary objects. The conceived trajectories of the human and the walls are depicted in Fig 8(d). Note that at certain locations (Fig 7(e) and 7(j)), the algorithm may fail to unhide occluded portions of the black wall due to concealing foreground (e.g. the human) and sensor noise.

Within the constraints of real environment, experiments were conducted in several indoor arenas (dark rooms, lounges, corridors) as well as in appropriate outdoor spaces. The algorithm remains successful in detecting, separating and tracking the environmental objects, as evident in Figs 7 and 8. These results exhibit the validity of the proposed algorithm in real-world scenarios.

### 4.3 Algorithm optimal performance

The results described in Sec. 4.1 and 4.2 consider different scenarios, environments, motion patterns of objects, trajectories to be followed, etc. This section explores the limiting cases of the proposed approach. We select suitable parameter values for the simulations; the static threshold is 0.02m, the dynamic threshold is 0.4m, and the size of objects is tested from 0.2m to 2m with the sensor scanning interval of 0.002 sec.

Due to the predefined thresholds, the algorithm better tracks objects moving in FoV with speed falling in a specific range. The accuracy may decrease for objects turning away from FoV. It is because our approach is feature agnostic, to mean that it does not recognize objects by their dimensions or other geometrical features. The size of objects is also restricted by the predefined clusters. An object containing less than 15 data points is simply discounted as sensor noise. On the other hand, 5000 data points are decomposed into multiple objects. Nevertheless, these thresholds are set according to the given environment.

**Fig 9:**
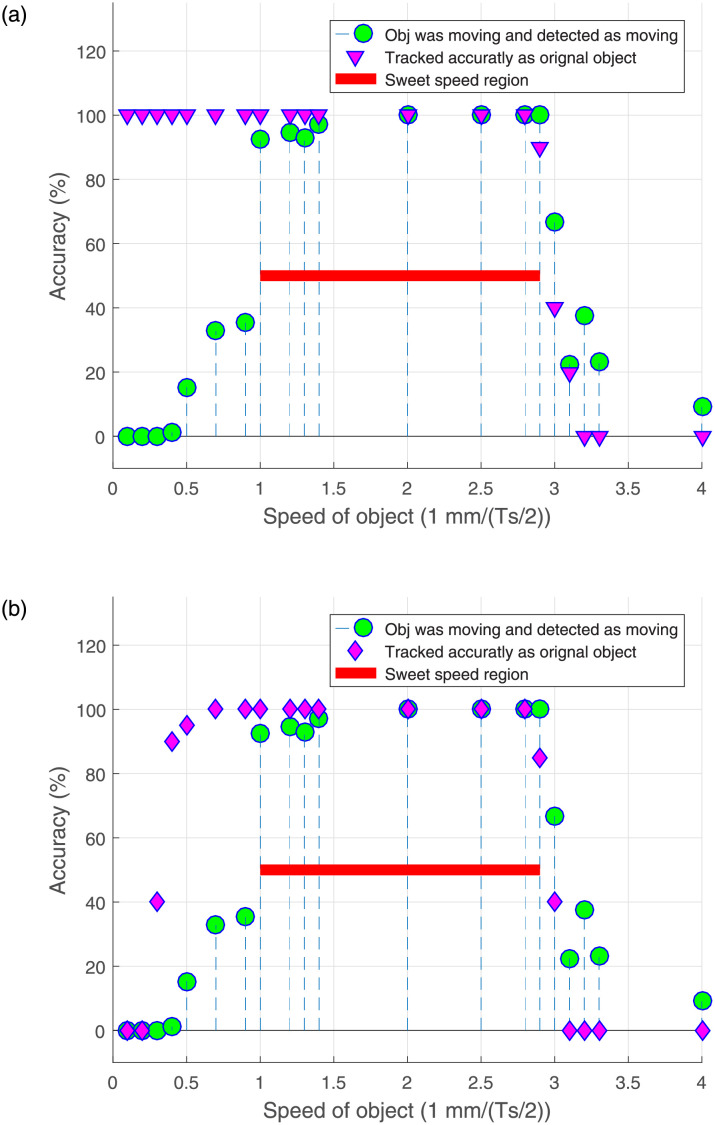
Boundaries of the approach.

Fig 9(a) illustrates the best detection accuracy w.r.t. the object speed during its motion inside FoV. The speed of the objects is presented in 1 mm/(Ts/2), where Ts represents the sensor sampling time set to 0.002 sec. The figure shows that when the speed of an object is less than 1 mm/(Ts/2), it is correctly detected as an original object, but incorrectly detected as static. For speed range 1 to 2.9 mm/(Ts/2), the approach successfully detects as well as accurately tracks the speed of an object. Therefore, this region is named as the sweet speed region. When the speed exceeds 2.9 mm/(Ts/2), the dynamic threshold is eclipsed and the object is treated as multiple objects. Note that tracking is only possible as long as an object remains identifiable.

Fig 9(b) deals with the objects returning to the FoV after a brief absence. In this case, detection and tracking are also speed-dependent. Therefore, if an object moves with a speed lower than 0.4 mm/(Ts/2), the algorithm may mistake it for a new object or fail to treat it as a dynamic object. For speed range 0.4 to 1 mm/(Ts/2), the approach considers dynamic objects as static upon reappearance, unless the object label was initialized to *dynamic*. Based on these observations, we establish that the sweet speed region is 1 to 2.9 mm/(Ts/2), as highlighted in Fig 9(b) with a solid line. At speed greater or equal to 3 mm/(Ts/2), the objects are not detectable, therefore untraceable.

Turning angle also tends to limit the algorithm’s capability of tracking objects following the curved paths. We observe that when the objects turn inside FoV, they are always accurately traceable. Accuracy declines when objects turn outside from FoV. In this experiment, the speed of the objects is set to 2 mm/(Ts/2). When objects take sharp turns angling 70° to 110° outside FoV, they are not correctly tracked. The geometry and dimensions of the obstacles, portion of an object falling outside FoV, irregular motion pattern before vanishing and other similar complex scenarios further hinder detection and tracking. The performance is restored when objects turn outside FoV at an angle range 0° to 79° or 111° to 180°.

## 5 Conclusions and discussion

The manuscript presents an algorithm for the detection, separation, and tracking of static and dynamic objects in an unknown environment. Our approach adopts a featureless scheme based on a 2D LiDAR sensor that decodes sensory data into likely time-varying boundaries of objects for their detection and tracking. It identifies the objects those are disappeared from the robot’s FoV and are reemerged at different locations. This is achieved by applying the local linear regression to the previously maintained histories of the objects. An overview of the proposed approach is summarized as follows.

Initially, sensory information is converted from data points to clusters using Euclidean distance based clustering. Each cluster represents a separate detected object. With the help of these clusters, the approach extracts the dimensions of each object by utilizing PCL. It includes center, minimum and maximum positions. The extracted parameters for each scan, saved in a history, play a vital role in tracking an object by iteratively updating its status (static or dynamic). Initially, the status of all objects is set to static while their identifiers are randomly chosen and the history is populated with the initial clusters. These variables are updated in the subsequent scans, wherein the objects are tracked in two steps. In the first step, objects which are static and moving inside FoV are tracked by comparing the Euclidean distance between the center points in consecutive scans. However, the objects which are newly detected or reappearing in FoV, are detected with the help of local linear regression in the second step. After some environmental scans, the approach accurately separates static and dynamic objects by tracing their history data.

Various V-Rep simulated environments quantify the performance of the algorithm. We assume that the sensory information is accurate and the robot parameters are obtained from the V-Rep built-in functions. Sensory data is transformed to a global frame for calculations. Robot Operating System (ROS) implements control routines for the robot. We also test the algorithm in real-world experimental environments and find that it outputs an accurate estimation of moving objects in the indoor arenas.

**Fig 10:**
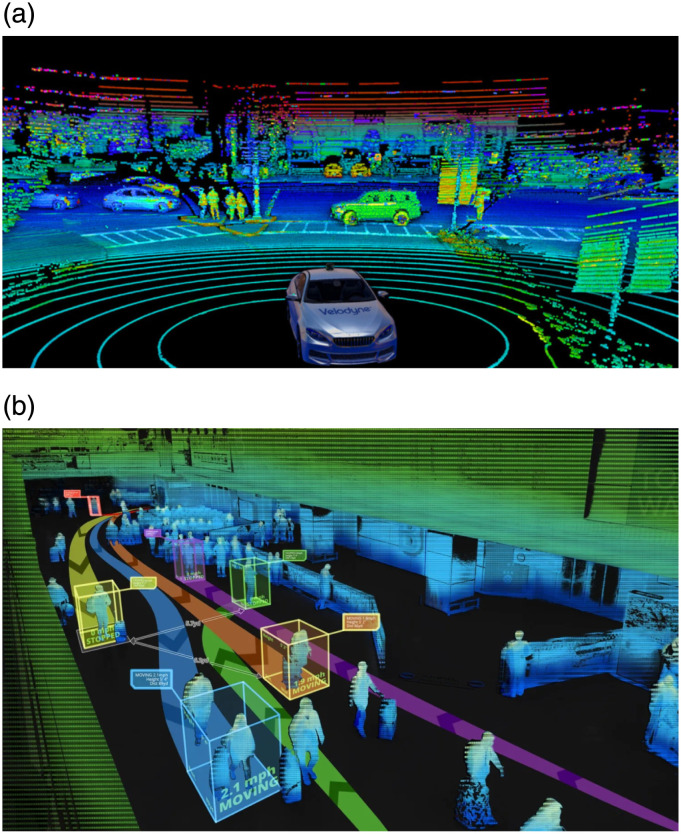
Application in real-world environment; (a) autonomous driving, (b) people tracking for security purpose.

An interesting avenue for future work lies in comparison with the probabilistic schemes for object detection and tracking. Our algorithm avoids lengthy computations of probabilistic methods by tracking threshold boundaries of the moving objects and consulting scan history. For what type of dynamic environments does the threshold boundaries approach becomes less effective, would seek further investigation. Another direction motivates the extension of the proposed scheme to the outdoor environments. After considering important parameters such as weather and lighting conditions and variations in the dimensions and speeds of the objects, we feel that a 3D LiDAR sensor providing an enhanced FoV, can improve detection accuracy which generally leads to higher tracking performance. Fig 10 shows two applications deploying 3D LiDAR sensors for objects (vehicle or human) detection. Lastly, the outputted map of the static environment can also be used for path planning and obstacle avoidance. Improved strategies of autonomous control can benefit industrial pick and place robots or collaborative surveillance agents [51–53].
